# *MicroRNA-215* as a Diagnostic Marker in Egyptian Patients with Hepatocellular Carcinoma

**DOI:** 10.31557/APJCP.2019.20.9.2723

**Published:** 2019

**Authors:** Hussein Ahmed El Mahdy, Ismail Abdelshafy Abdelhamid, Ahmed Ibrahim Amen, Eman Abdelsameea, Mona M Hassouna

**Affiliations:** 1 *Department of Biochemistry, *; 2 *Department of Chemistry, Faculty of Science, Cairo University,*; 3 *Department of Hepatology and gastroenterology,*; 4 *Department of Clinical pathology, National Liver Institute, Menoufia University, Egypt. *

**Keywords:** hepatocellular carcinoma (HCC), chronic hepatitis C virus (chronic HCV), MicroRNA-215 (miR-215)

## Abstract

**Background:**

*MicroRNAs* are mentioned as a small non-coding RNAs groups and aberrant *miRNA* expression was found in hepatocellular carcinoma (HCC) patients.

**Aim:**

To evaluate role of plasma MicroRNA-215 as a diagnostic tool in HCC patients.

**Methods:**

A prospective study included 195 subjects: healthy controls (group I), cirrhotic patients (group II), and patients with HCC (group III). Clinical examination, radiological and laboratory investigations which included quantification of *miR-215* by Real-time qPCR were done for all cases.

**Results:**

Spearman’s rank correlation revealed that in HCC group, there was a negative correlation between *MiRNA-215* and serum AFP levels and focal size lesion (cm) (rs = -0.72, - 0.94 respectively, p<0.001). Receiver operating characteristics analysis for discrimination between cirrhosis and HCC groups regarding microRNA-215 displayed 78.3% sensitivity, 88.0% specificity at cutoff value of ≤ 1.90. Area under the curve (AUC) was 0.87 (p< 0.001). As regards AFP, it had a sensitivity of 81.7%, a specificity of 66.7 at cutoff value of ≥ 11.50 (ng/mL).

**Conclusions:**

Plasma level of miR-215 may be a promising biomarker in HCC diagnosis. Moreover, if *miR-215 *combined with AFP, it can be used as a diagnostic biomarker, for early detection of HCC.

## Introduction

Hepatocellular carcinoma (HCC) is one of the common cancers worldwide as it represents the 5th one in men and the 7^th^ one in women (Bosetti et al., 2014) and the 3^rd^ one of mortality related tumors. Most patients with hepatic cirrhosis may develop HCC and finally death may occur (Salgia and Singal, 2014). The prognosis of HCC still being unsatisfactory in spite of development in surgical and non-surgical treatments compared to other cancer tumors (W Pang and TP Poon, 2012).

Number of HCV patients worldwide is in the range of 170 to 200 million. Genotype 4 is the most commonly HCV genotype in Egypt and it represents about 90% of cases, research is not enough, because its localization is restricted to Africa and Middle East (Shelbaya et al., 2015). Significant numbers of infected patients are at high risk of developing liver cirrhosis and subsequently hepatocellular carcinoma. About 10% to 15% of HCC cases are resecTablebecause of the late diagnosis.

*MicroRNAs* (*miRNAs*) are one of evolutionarily conserved small non-coding RNAs which make a contribution in the gene expression regulation and protein translation, and incriminated in differentiation, cell evolution, and occurrence of diverse types of cancers. miRNAs are released to extracellular areas, and they are extremely constant in body fluids, which includes plasma or serum, in which they are packaged into several microparticles or related with RNA-binding proteins because of passive leakage that caused by the deceased cell, and also they are actively secreted through exosomes from cells (Aravalli et al., 2013; Li et al., 2015). Different researches have confirmed that circulating miRNAs could be considered as a biomarker for HCC prognosis and diagnosis (Zhang et al., 2015).

Sun et al., (2013) reported that, deregulation of various miRNAs in exclusive types of cancer such as HCC. 

It is common to found down regulation of *miRNAs *subsets as some types of them may act as assumed tumor suppressor genes in HCC. Tumor suppressive* miRNAs *restorations blocks cell cycle, increased apoptosis, decrease tumor angiogenesis and metastasis by stopping migration and invasion. Of these *miRNAs*,* miR-122* *miR-199*, and *miR-215* appear to be particularly important in HCC (Hou et al., 2011; Ashmawy et al., 2017). *MiRNAs *control expression of many target genes in HCC and their profiling reveals molecular mechanisms of pathogenesis and hidden visions into detection and treatment of HCC (Song He, 2015). Our aim was to evaluate role of plasma miR-215 as a diagnostic tool in Egyptian patients with HCC.

## Materials and Methods

Our research was done in hepatology and gastroenterology department at the National Liver Institute, Menoufia University in the period from November 2016 till December 2017. It included 135 patients and 60 healthy subjects serving as control group. We have 3 groups:

*Group I (Control)*

This group included 60 apparently healthy individuals. They were 32 males and 28 females, whose ages ranged from 39 to 67 years old (mean ±SD= 51.67 ± 6.40 years).

*Group II (Cirrhosis)*

This group included 75 patients with liver cirrhosis due to chronic HCV infection. They were 41 males and 34 females whose ages ranged from 41 to 68 years old (mean ±SD= 54±6.73 years). 

*Group III (HCC)*

Sixty patients with HCC were included. They were 35 males and 25 females, whose ages ranged from 41 to 70 years old (mean ±SD= 53.97±6.15 years). 

All studied subjects provided informed written consent and the study approval by local ethics committee of National Liver Institute, Menoufia University was obtained. 

*Exclusion criteria*

All patients with the following: hepatitis B virus (HBV) or HIV, immunosuppression, organ transplantation, autoimmune disease, Schistosomiasis, all other malignancies and lastly patients who were under chemotherapy and antiviral treatment were excluded. 

All individuals were subjected to complete clinical history and examination, laboratory investigations and abdominal ultrasonography.

*Radiological study*

Abdominal ultrasound was done to assess liver size, coarseness of parenchyma, liver surface nodularity, lymph nodes enlargement and size, spleen size (which if enlarged can suggest portal hypertension), patency and flow of veins and arteries and focal lesions (which if present can suggest hepatocellular carcinoma).

All HCC patients were diagnosed by characteristic vascular enhancement pattern detected by multislice triphasic spiral CT scan or MRI according to established diagnostic criteria (Galle et al., 2018).

*Laboratory Investigations*

- Complete blood picture was determined by the Sysmex^®^ Automated Hematology Analyzer KX-21N (Sysmex Corporation, Kobe 651-0073, Japan). Liver tests: aspartate transaminase (AST) and alanine transaminase (ALT), serum albumin, total and direct bilirubin, alkaline phosphatase and INR were measured. Creatinine level in serum was measured in (mg/dl). The analysis of serum alpha fetoprotein (AFP) (ng/ml) was done by IMMULITE 1000 system supplied by Siemens kit (SIEMENS Medical Solutions Diagnostics, USA). Chronic HCV infection was diagnosed by HCV antibodies detection and also HCV RNA level detection by real time PCR. Anti-HCV antibodies were founded by 3^rd^ generation enzyme immunoassay (Ortho HCV version 3.0 ELISA; Ortho- Clinical Diagnostics INC., Raritan. NJ, USA). Quantification of HCV RNA level was performed by COBAS Taqman 84 (Roche) real time HCV RNA assay with lower detection limit 15 IU/ml. 

- Model of End-Stage Liver Disease (MELD): It was measured according to next formula: MELD =3.78×ln [serum bilirubin (mg/dL)] + 11.2×ln [INR] + 9.57×ln [serum creatinine (mg/dL)] + 6.43 (Malinchoc et al., 2000). 

MiRNA Quantification by Real-Time qPCR (RT-qPCR): Blood samples were collected in EDTA tubes, centrifuged at 4,000 rpm for 10min at 4^o^C, plasma separated, and stored at −70^o^C.

*RNA Extraction and cDNA Synthesis*

Total RNA was taken from 200 µl plasma by the miRNeasy extraction kit (Qiagen, Germany). RNA purity was evaluated by the RNA concentration and quantified by Nano Drop Spectrophotometer (NanoDrop ND-1000, United States). Reverse transcription was done for cDNA synthesis using miRNeasy Plasma Reverse Transcription Kit (Qiagen, Germany) regarding to manufacturer’s instructions.

*Amplification and Quantification*

The expressions miR-215 was evaluated by RT-qPCR analysis regarding to manufacturer’s protocol. The housekeeping *miRNAU6* (*RNU6*) was used as the endogenous control. For qRT-PCR, 2 μL of diluted reverse transcription products was mixed with 12.5 μL SYBR^®^ Green Real-time PCR Master Mix (Qiagen, Germany), 2.5 μL of forward primer, 2.5 μL reverse primers and 5 μL RNase-free water (in syper green box) in a final volume of 25 μL in each strip tube according to the manufacturer’s instructions. The reaction was performed on AB Applied Biosystem Real-Time System (Model: 7500 system, USA), with the following reaction conditions: 95°C for 15 min, followed by 40 cycles at 94°C for 15 sec, 55 °C for 30 sec, and 70°C for 34 sec. 

Array plate for PCR was full of reverse and forward miRNA-specific primers.

The primer sequences were used for miR-215: forward primer: 

**Figure:**
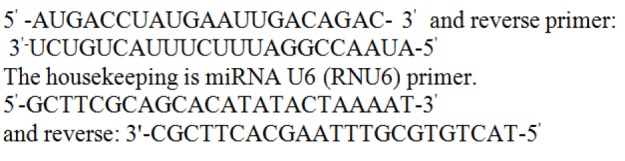


*Statistical analysis*

Results were analyzed by using statistical package of social sciences (SPSS 22.0, IBM/SPSS Inc., Chicago, IL). The mean ± standard deviation (SD) was used for normally distributed data whereas interquartile range (IQR) and median was for skewed data. Categorical data was demonstrated as frequency with percentage. For continuous variables, ANOVA test was used to compare between several groups when homogeneity and normality assumptions were met, instead, its non-parametric equivalent Kruskal-Wallis test was applied upon violation. For multiple pairwise comparisons, an appropriate Post Hoc test was used as Tukey-HSD test with significant ANOVA or Bonferroni test with significant Kruskal-Wallis test. The Chi-square (*X*^2^) test was used to compare categorical variables. Binary logistic regression analysis was used for deriving the best fitting models that describe relation between presence of HCC and combined biomarkers measurement. We used Receiver operating characteristic analysis for assessment of diagnostic performance of plasma miR-215 in HCC detection. The p-values < 0.05 were considered as significant ones. 

## Results

This study included 135 patients who were selected from outpatient clinics and inpatient unit of hepatology and gastroenterology department of the National Liver Institute, Menoufia University and were classified into 2 groups, 75 cirrhotic patients (cirrhosis group) and 60 patients with HCC (HCC group) and control group which included 60 healthy volunteers with matched age and gender. 

Regarding age and gender distribution among studied groups as shown in Table 1, there was no statistically significant difference among those group (p= 0.071, 0.848 respectively).

Descriptive and analytical statistics of liver tests and serum creatinine in all groups were represented in Table 2. There was significant difference between cirrhosis and HCC groups as regards ALP (p=0.004), ALT, AST and GGT (p< 0.001). 

In control group, *miRNA-215* ranged from 1.60 to 21.30 with a median value of 6.89. In cirrhotic patients group, it ranged from 0.70 to 14.65 with a median value of 2.85. In patients with HCC group, it ranged from 0.03 to 10.95 with a median value of 0.52. *MiRNA-215* and AFA mean levels showed a statistically significant difference (p <0.001) between the three studied groups (Table 3).

**Figure 1:**
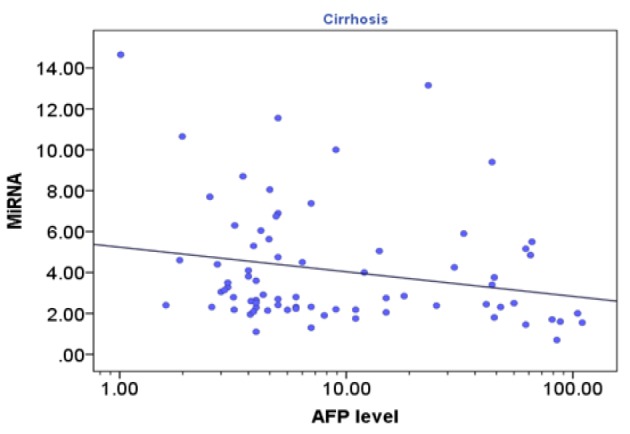
Correlation between Circulating microRNA-215 and AFP Serum Level (log10 scaled) in Cirrhosis Group (rs= -0.27; p<0.020)

**Figure 2:**
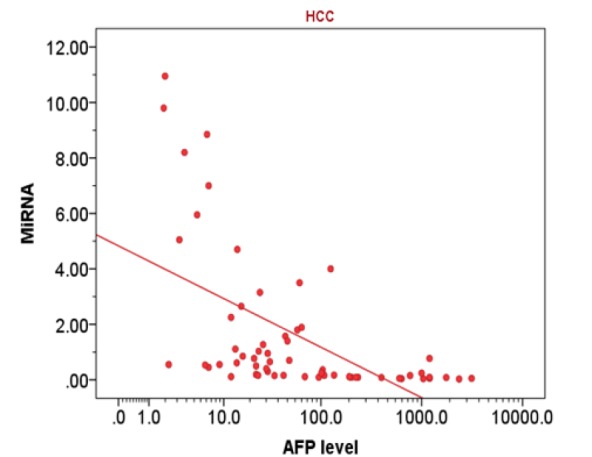
Correlation between Circulating Microrna-215 and AFP Serum Level (Log10 Scaled) in HCC Group

**Figure 3:**
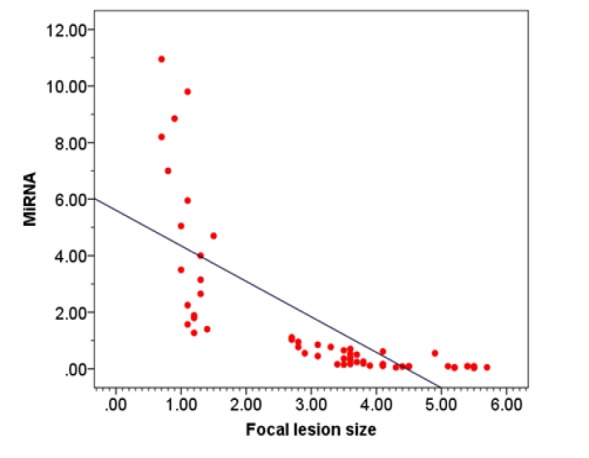
Correlation between Circulating microRNA-215 and Focal Size of Focal Lesion in HCC Group (R= - 0.94, p< 0.001)

**Figure 4:**
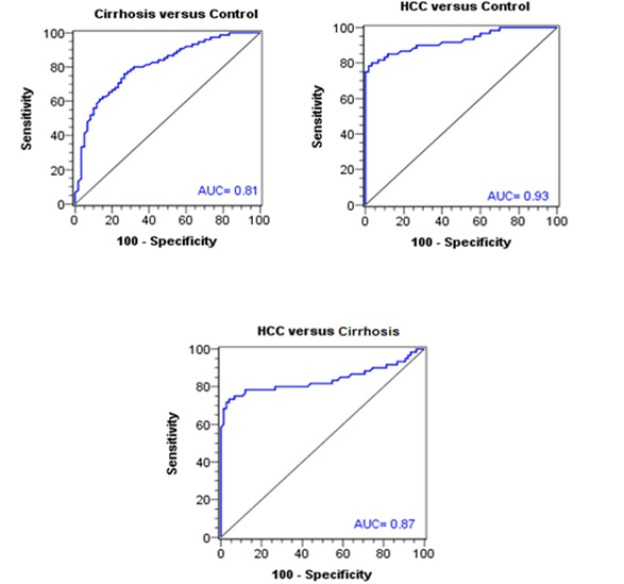
ROC Curves of Circulating miRNA-215 for Pairwise Dicrimination between the Three Studied Groups

**Table 1:** Age and Gender Distribution among Studied Groups

Age (years)	GI	GII	GIII	significant test	p-value
	Control (n = 60)	Cirrhosis (n = 75)	HCC (n = 60)		
Median (IQR)	51.0 (8.0)	53.0 (9.0)	54.0 (8.0)	*F* = 2.68^a^	0.071 ^NS^
Mean ± SD	51.67 ± 6.40	54.00 ± 6.73	53.97 ± 6.15		
Range (min-max)	39.00 - 67.00	41.00 - 68.00	41.00 - 70.00		
Gender [n (%)]					
Male	32 (53.3)	41 (54.7)	35 (58.3)	*x* ^2^ * = *0.33^b^	0.071 ^NS^
Female	28 (46.7)	34 (45.3)	25 (41.7)		

NS, Non significant at p-value ≥ 0.05; IQR, Interquartile range; n, Number; SD, Standard deviation; a, ANOVA test; b, Chi-Square test

**Table 2:** Laboratory Investigations among the Studied Groups

Parameters	GI	GII	GIII	Test of significance	Post hoc test
	Control (n = 60)	Cirrhosis (n = 75)	HCC (n = 60)	(Kruskal- Wallis)	
ALT (U/L)					
Median (IQR)	16.00 (7.75)	30.00 (39.0)	62.00 (29.75)	*x* ^2^ = 93.81	p_1_< 0.001^HS^
Mean ± SD	17.08 ± 5.55	41.72 ± 35.02	63.45 ± 22.33	*p-value*	p_2_< 0.001^HS^
Range (min-max)	8.00 - 33.00	8.00 - 200.0	22.00 - 119.00	<0.001^HS^	p_3_< 0.001^HS^
AST (U/L)					
Median (IQR)	19.00 (4.75)	37.00 (47.00)	63.00 (26.00)	*x* ^2^ = 86.65	p_1_< 0.001^HS^
Mean ± SD	18.58 ± 3.77	49.24 ± 36.75	65.05 ± 26.72	*p-value*	p_2_< 0.001^HS^
Range (min-max)	10.00 - 30.00	9.00 - 180.00	20.00 - 170.00	<0.001^HS^	p_3_< 0.001^HS^
ALP (U/L)					
Median (IQR)	63.50 (18.75)	98.00 (33.00)	121.50 (52.75)	*x* ^2^= 96.29	p_1_< 0.001^HS^
Mean ± SD	61.47 ± 10.51	98.65 ± 26.85	122.53 ± 35.03	*p-value*	p_2_< 0.001^HS^
Range (min-max)	41.00 - 80.00	46.00 - 148.00	55.00 - 197.00	<0.001^HS^	p_3_= 0.004 ^HS^
g GT (U/L)					
Median (IQR)	20.00 (7.00)	42.00 (39.00)	80.00 (66.00)	*x* ^2^ = 65.39	p_1_< 0.001^HS^
Mean ± SD	19.50 ± 5.97	43.80 ± 21.71	76.35 ± 40.06	*p-value*	p_2_< 0.001^HS^
Range (min-max)	9.00 - 39.00	15.00 - 105.00	18.00 - 169.00	<0.001^HS^	p_3_< 0.001^HS^
Total bilirubin (mg/dL)					
Median (IQR)	0.54 (0.32)	1.30 (2.10)	1.28 (1.19)	*x* ^2^ = 71.08	p_1_< 0.001^HS^
Mean ± SD	0.57 ± 0.19	1.87 ± 1.55	1.91 ± 1.65	*p-value*	p_2_< 0.001^HS^
Range (min-max)	0.20 - 0.90	0.20 - 7.27	0.60 - 7.50	<0.001^HS^	p_3_= 0.360 ^NS^
Direct bilirubin (mg/dL)					
Median (IQR)	0.10 (0.06)	0.70 (1.50)	0.40 (0.62)	*x* ^2^ = 94.83	p_1_< 0.001^HS^
Mean ± SD	0.12 ± 0.06	1.07 ± 1.03	0.80 ± 0.97	*p-value*	p_2_< 0.001^HS^
Range (min-max)	0.01 - 0.25	0.10 - 4.60	0.10 - 4.40	<0.001^HS^	p_3_= 1.000 ^NS^
Albumin (g/dL)					
Median (IQR)	4.30 (0.75)	3.00 (1.80)	3.45 (0.80)	*x* ^2^ = 64.28	p_1_< 0.001^HS^
Mean ± SD	4.33 ± 0.43	3.23 ± 0.97	3.40 ± 0.56	*p-value*	p_2_< 0.001^HS^
Range (min-max)	3.60 - 5.10	1.60 - 4.90	2.10 - 4.40	<0.001^HS^	p_3_= 1.000 ^NS^
Platelets (10^3^ /mL)					
Median (IQR)	266.00 (91.75)	151.00 (95.00)	108.00 (61.75)	*x* ^2^ = 12.47	p_1_= 0.001^HS^
Mean ± SD	272.83 ± 49.63	172.77 ± 82.05	118.92 ± 65.57	*p-value*	p_2_< 0.407 ^NS^
Range (min-max)	184.00 - 360.00	39.00 - 333.00	22.00 - 340.00	=0.002 ^HS^	p_3_= 0.160 ^NS^
Creatinine (mg/dL)					
Median (IQR)	0.81 (0.29)	0.90 (0.35)	1.00 (0.30)	*x* ^2^ = 12.19	p_1_< 0.030^S^
Mean ± SD	0.84 ± 0.17	0.96 ± 0.27	0.97 ± 0.20	*p-value*	p_2_< 0.002 ^HS^
Range (min-max)	0.50 - 1.24	0.60 – 1.90	0.60 - 1.60	<0.002^HS^	p_3_ = 0.997^NS^

a, Multiple pairwise comparisons by Post hoc test; -p_1_:p-value for the difference between control and cirrhosis group (GI vs. GII); p_2_, p-value for the difference between control and HCC group (GI vs. GIII); p_3_, p-value for the difference between cirrhosis and HCC group (GII vs. GIII); IQR, Interquartile range; NS, Non significant at p-value ≥ 0.05; S, Significant at p-value < 0.05; HS, Highly significant at p-value < 0.01

**Table 3:** The Median of microRNA -215 and AFP among the Studied Groups

Parameters	GI	GII	GIII	Test of significance Kruskal-Wallis	Post hoc test
	Control	Cirrhosis	HCC		
	(n = 60)	(n = 75)	(n = 60)		
MiRNA-215					
Median (IQR)	6.89 (7.33)	2.85 (2.96)	0.52 (1.63)	*x* ^2^ = 93.42	p_1_< 0.001^HS^
				*p-value*	p_2_< 0.001^HS^
Range (min-max)	1.60 - 21.30	0.70 - 14.65	0.03 - 10.95	<0.001^HS^	p_3_< 0.001^HS^
AFP (ng/mL)					
Median (IQR)	3.95 (2.53)	5.50 (21.20)	38.00 (184.76)	*x* ^2^ = 69.63	p_1_= 0.001^HS^
				*p-value*	p_2_< 0.001^HS^
Range (min-max)	0.80 - 10.50	1.01 - 110.00	1.80 - 3130.00	<0.001^HS^	p_3_< 0.001^HS^

a, Multiple pairwise comparisons by Post hoc test; -p_1_, p-value for the difference between control and cirrhosis group (GI vs. GII); -p_2_, p-value for the difference between control and HCC group (GI vs. GIII); p3, p-value for the difference between cirrhosis and HCC group (GII vs. GIII); IQR, Interquartile range; NS, Non significant at p-value ≥ 0.05; S, Significant at p-value < 0.05; HS, Highly significant at p-value < 0.01.

**Table 4:** ROC Curves of Circulating miRNA-215 for Pairwise Dicrimination between the Three Studied Groups

Outcome Disease	Model Variables	Coefficient	SE	Wald Square	*p-value* ^a^	OR (95% CI)
Cirrhosis vs. control	AFP (log_10_)	2.18	0.72	9.18	0.002 ^HS^	8.82 (2.16-36.03)
	miRNA (log_10_)	-3.98	0.85	21.74	< 0.001 ^HS^	0.02 (0.003-0.10)
	Constant	1.41	0.79	3.18	0.074 ^NS^	-
	Equation	Logit (p)*= 2.18 × (log_10_ AFP) - 3.98 × (log_10_ miRNA) +1.41				
	*x* ^2^- value	54.92				
	*p-value* ^b^	< 0.001 ^HS^				
HCC vs. control	AFP (log_10_)	3.21	1.1	8.59	0.003 ^HS^	24.83 (2.9-212.72)
	miRNA (log10)	-3.01	0.91	10.86	0.001 ^HS^	0.05 (0.01-0.29)
	Constant	-1.44	1.17	1.51	0.219 ^NS^	-
	Equation	Logit(p)*= 3.21 × (log_10_ AFP) - 3.01 × (log_10_ miRNA) - 1.44				
	*x* ^2^- value	105.11				
	*p-value* ^b^	< 0.001 ^HS^				
HCC vs. cirrhosis	AFP (log10)	0.55	0.43	1.61	0.204 ^NS^	1.74 (0.74-4.06)
	miRNA (log10)	-2.89	0.64	20.38	< 0.001 ^HS^	0.06 (0.02-0.20)
	Constant	-0.29	0.64	0.21	0.646 ^NS^	-
	Equation	Logit(p)*= 0.55 × (log_10_ AFP) - 2.89 × (log_10_ miRNA) -0.29				
	*x* ^2^- value	67.87				
	*p-value* ^b^	< 0.001 ^HS^				

Predicted probability: p=1/(1+e^(-logit(p)) ); * logit(p)=ln(p/(1-p));* x*^2^ value, model chi square; ^a^, p-value of Wald test squared; ^b^, p-value corresponds to overall chi square of the model; NS, Non significant at p-value ≥ 0.05; S, Significant at p-value < 0.05; HS, Highly significant at p-value < 0.01; OR (95% CI), Odds ratio with 95 % confidence interval

**Table 5:** Logistic Regression Models of Combined Circulating microRNA-215 and AFP Measurements

Outcome Disease	Model Variables	Coefficient	SE	Wald Square	*p-value* ^a^	OR (95% CI)
Cirrhosis vs. control	AFP (log_10_)	2.18	0.72	9.18	0.002 ^HS^	8.82 (2.16-36.03)
	miRNA (log_10_)	-3.98	0.85	21.74	< 0.001 ^HS^	0.02 (0.003-0.10)
	Constant	1.41	0.79	3.18	0.074 ^NS^	-
	Equation	Logit (p)*= 2.18 × (log_10_ AFP) - 3.98 × (log_10_ miRNA) +1.41				
	*x* ^2^- value	54.92				
	*p-value* ^b^	< 0.001 ^HS^				
HCC vs. control	AFP (log_10_)	3.21	1.1	8.59	0.003 ^HS^	24.83 (2.9-212.72)
	miRNA (log10)	-3.01	0.91	10.86	0.001 ^HS^	0.05 (0.01-0.29)
	Constant	-1.44	1.17	1.51	0.219 ^NS^	-
	Equation	Logit(p)*= 3.21 × (log_10_ AFP) - 3.01 × (log_10_ miRNA) - 1.44				
	*x* ^2^- value	105.11				
	*p-value* ^b^	< 0.001 ^HS^				
HCC vs. cirrhosis	AFP (log10)	0.55	0.43	1.61	0.204 ^NS^	1.74 (0.74-4.06)
	miRNA (log10)	-2.89	0.64	20.38	< 0.001 ^HS^	0.06 (0.02-0.20)
	Constant	-0.29	0.64	0.21	0.646 ^NS^	-
	Equation	Logit(p)*= 0.55 × (log_10_ AFP) - 2.89 × (log_10_ miRNA) -0.29				
	*x* ^2^- value	67.87				
	*p-value* ^b^	< 0.001 ^HS^				

Predicted probability: p=1/(1+e^(-logit(p)) ); * logit(p)=ln(p/(1-p));* x*^2^ value, model chi square; ^a^, p-value of Wald test squared; ^b^, p-value corresponds to overall chi square of the model; NS, Non significant at p-value ≥ 0.05; S, Significant at p-value < 0.05; HS, Highly significant at p-value < 0.01; OR (95% CI), Odds ratio with 95 % confidence interval

**Table 6:** Test Characteristics of Combined Tests of miRNA-215 and AFP

Test characteristics	Combined Tests		
	Cirrhosis vs. Control	HCC vs. Control	HCC vs. Cirrhosis
Best cutoff value	≥ 0.56	≥ 0.52	≥ 0.47
AUC	0.85	0.94	0.85
SE	0.03	0.03	0.04
p-value	< 0.001^HS^	< 0.001^HS^	< 0.001^HS^
Sensitivity %	76	88.3	78.3
Specificity %	81.7	98.3	90.7
PPV %	83.8	98.1	87
NPV %	73.1	89.4	84
Accuracy %	78.8	93.3	84.5

SE, Standard error; PPV, Positive predictive value; NPV, Negative predictive value; NS, Non significant at p-value ≥ 0.05; S33, Significant at p-value < 0.05; HS, Highly significant at p-value < 0.01

**Table 7:** Statistical Analysis of miRNA-215 Regarding Focal Lesion Number in HCC Group

MiRNA	Single	Multiple	Mann-Whitney test	*p-value*
Median (IQR)	3.75 (5.43)	0.17 (0.46)	Z = 6.10	< 0.001^HS^
Mean ± SD	4.67 ± 3.11	0.33 ± 0.31		
Range (min-max)	1.27 - 10.95	0.03 - 1.11		

**Table 5:** Logistic Regression Models of Combined Circulating microRNA-215 and AFP Measurements

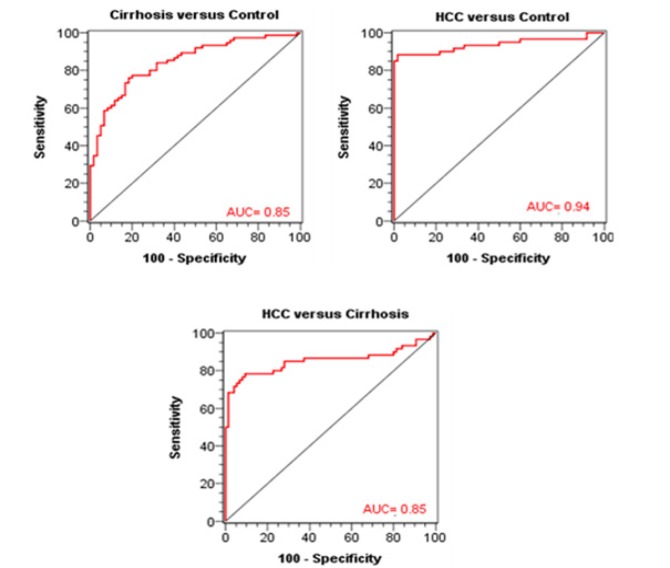

Spearman’s rank correlation revealed that in cirrhosis group, there was a negative correlation between *miRNA-215* level and serum AFP level (rs= -0.27; p<0.020) as illustrated in Figure 1 and in HCC group, there was a negative correlation between *MiRNA-215* level and serum AFP level, (rs= -0.72; p<0.001) as illustrated in Figure 2. Also, there was a negative correlation between *miRNA-215* level and focal size lesion (cm) in HCC group (rs = - 0.94, p< 0.001) as shown in Figure 3. 

Regarding MELD score, there was no correlation between miRNA-215 level and MELD score in both cirrhosis (rs = -0.13, p=0.261) and HCC (rs = -0.01, p=0.921) groups.

Receiver operating characteristics (ROC) analysis was used to assess the diagnostic performance of circulating microRNA-215 and AFP in HCC, individually and combined. For discrimination between cirrhosis and HCC groups regarding microRNA-215, a discrimination was displayed with 78.3% sensitivity, 88.0% specificity, 83.9% PPV, 83.5% NPV, and 83.2% accuracy at the best cutoff value of ≤ 1.90. The area under the curve (AUC) was 0.87 with SE of 0.04 (p-value < 0.001) (Table 4 and Figure 4). As regards AFP, it had a sensitivity of 81.7%, a specificity of 66.7%, a PPV of 66.2%, a NPV of 82.0% and an accuracy of 74.17% at the best cutoff value of ≥ 11.50 (ng/mL). The AUC was 0.77 with SE of 0.04 (p-value < 0.001) (Table 4).

Binary logistic regression analysis was conducted to evaluate the contribution of combined measurement of miRNA-215 and AFP to assign individuals to the outcome disease whether cirrhosis or HCC. 

Table 5 showed variables included in the models, the estimated equation derived from each model, and models Chi square values with the associated p-values indicating the overall significant (p <0.001) fit of the models. 

In the first model, the outcome disease is cirrhosis against control, the p-values for the regression coefficients relevant to log10 AFP (p= 0.002, OR = 8.82, 95% CI: 2.16-36.03) and log10 miRNA-215 (p < 0.001, OR = 0.02, 95% CI: 0.003-0.10) revealed that both biomarkers contributed significantly to assign patients to cirrhosis group. For the second model, the outcome disease is HCC against control. The p-values for the regression coefficients of log10 AFP (p= 0.003, OR = 24.83, 95% CI: 2.90-212.72) and log10 miRNA-215 (p =0.001, OR = 0.05, 95% CI: 0.01-0.29) indicated that both biomarkers contributed significantly to assign patients to HCC group. 

In the third model, the outcome disease is HCC against cirrhosis, the p-value associated with the regression coefficients of log10 *miRNA-215* (p <0.001, OR = 0.06, 95% CI: 0.02-0.20) indicated a highly significant contribution of *miRNA-215* measurement. However, log10 AFP (p= 0.204, OR = 1.74, 95% CI: 0.74 - 4.06) revealed non-significant contribution of AFP measurement indicating superiority of* miRNA-215* over AFP regarding HCC prediction versus cirrhosis.

The characteristics of the combined *MiRNA-215* and AFP (both were log10 transformed) were illustrated in Table6 and graphically by ROC curves in figure 5. For discrimination between control and HCC, the estimates were 88.3% sensitivity, 98.3% specificity, 98.1% PPV, 89.4% NPV, and 93.3% accuracy at the best cutoff value of ≥ 0.52(ng/mL). The AUC was 0.94 with SE of 0.03 (p-value < 0.001).

Between cirrhosis and HCC groups, the ROC analysis estimated a sensitivity of 78.3%, a specificity of 90.7%, a PPV of 87.0%, a NPV of 84.0% and an accuracy of 84.5% at the best cutoff value of ≥ 0.47 (ng/mL). The AUC was 0.85 with SE of 0.04 (p-value < 0.001).

The mean ± SD of serum MiRNA-215 was significantly lower in patients with hepatocellular carcinoma who had multiple focal lesions than in those with single focal lesion (p< 0.001) as shown in Table 7.

## Discussion

The HCC incidence is rising all through the world because of a rising incidence of HBV and HCV infection, due to an increase in prevalence of non-alcoholic fatty liver disorder (Njei et al., 2015). In Egypt, HCC is the fourth common cancer and is the second reason of most cancers mortality in both sexes (Demerdash et al., 2017). The burden of HCC increased with a doubling within the occurrence rate in the past 10 years and this was attributed to excessive occurrence of HCV. Egypt has incidence of HCC approximately 21% in cirrhotic sufferers. 

Serum AFP measurement remains combined with ultrasound to reduce the hazard of missing small lesions within the cirrhotic liver that were not detected via ultrasound. Alternative or additional biomarkers could be beneficial tools for surveillance or as a decisional device in medical practice to discover patients that will benefit from advanced imaging techniques in a surveillance setting to augment the percentage of patients presented with HCC diagnosed in an early tumor stage (Schütte et al., 2015).

As for the relationship among *miRNAs* and HCC, several research have proven that the atypical miRNAs expression can be found in plasma and serum of HCC patients or HCC cells and tissues, and miRNAs have proven great promise as prognostic and diagnostic markers for HCC (Morishita and Masaki, 2015; Qi et al., 2013; Demerdash et al., 2017).

We found that the mean age was over 50 years old in both cirrhosis and HCC groups. Estes et al., (2015) reported that anti-HCV prevalence in Egypt was totally 14.7%, and prevalence of viremia was 9.8%. Prevalence in patients aged >59 years was similar to prevalence in those with age between 55 and 59 years.

Regarding the liver profile ALT, AST, GGT, total and direct bilirubin and ALP were higher in HCC and cirrhotic patients than in control group. In addition, Albumin values were lower to a significant level in HCC and cirrhotic patients than in control group. Our results agreed with Awadallah et al., (2011) who reported a significant deterioration in the liver function in cirrhosis and HCC compared to control group. However, there was no significant difference between HCC and cirrhotic groups regarding total and direct bilirubin and albumin results. Serum ALT, AST, GGT and ALP was higher in HCC patients compared to cirrhotic patients. Our findings were in accordance to Sleisenger et al., (2002) and Thapa and Walia ( 2007) who declared that aminotransferases are the most frequently utilized indicators of hepatocellular necrosis and expected to be elevated in liver cell injury whatever the cause and the conventional tests of hepatic function don’t distinguish HCC from cirrhosis and so they contribute little to the diagnosis of the tumor with the exception of serum ALP, which was significantly elevated in HCC group when compared with cirrhotic group due to the more prominent cholestatic effect of the tumor.

We found a significantly higher levels of AFP in HCC patients (median= 294.9 ng/mL) compared to the cirrhosis patients (median= 19.5 ng/mL) and control group (median= 4.3ng/mL). This was in agreement with Spadaro et al.; El-Assaly et al. and Awadallah et al. who reported a significant elevation in serum AFP in HCC group compared to cirrhosis patients and control group (Spadaro et al., 2005; El-Assaly et al., 2008; Awadallah et al., 2011).

Our results revealed a significantly lower levels of *MicroRNA-215* in HCC patients (median= 1.63) compared to the cirrhosis patients (median= 4.0) and control group (median= 8.6). This was in agreement with Ashmawy et al., (2017) who reported a significant lower expression of *MicroRNA-215* level in HCC group when compared to cirrhotic group and control group (Ashmawy et al., 2017). 

Significantly high correlation found between tumor grades and miR-215 level was consistent with numerous studies indicating its role in tumor progression. Similarly, we found that mean ± SD of serum *MiRNA-215 *was significantly lower in HCC patients who had multiple focal lesions than in those with single focal lesion (p< 0.001) and also, there was a negative correlation between *miRNA-215* level and focal lesion size (cm) in HCC group. 

Our results was not in agreement with Zhang et al., (2014) who reported a significant higher level of MicroRNA-215 in HCC group compared to cirrhosis patients and control group and the expression of miR-215 in plasma was significantly up-regulated in patients with chronic hepatitis and HCC. This is may be happened by genotypes. Demerdash et al., (2017) reported that about 90% of Egyptian patients suffering from HCV belong to genotype-4 and this differs from other countries at which HCC patients suffering from other genotypes.

The cut-off value of AFP for discriminating HCC patients versus cirrhosis and control groups was 8.9 ng/mL. This had a diagnostic sensitivity of 83.3%, specificity 95%. The cut-off value of AFP for HCC diagnosis has been a matter of debate. AFP had a specificity of 76%-94% and a sensitivity of 39%-65% for the presence of HCC in previously published research (Trevisani et al., 2001). The variation in specificity and sensitivity of AFP in the research may be because of the variety of patient populations who were examined, various study designs and different normal cut-off levels (Gomaa et al., 2009) .

Serum AFP is related to different problems; First, the rise in AFP level which occurred transiently in patients with chronic liver disease especially during exacerbation of hepatitis (serum level >100ng/mL) (low specificity) (Kobeisy et al., 2012), mild rise in acute hepatitis, chronic hepatitis and cirrhosis and overlaps can cause diagnostic difficulties. The second one is that AFP levels may be normal in up to 40% of patients with HCC, especially for the duration of the early stages (low sensitivity) (El-Assaly et al., 2008). However, El-Assaly et al. suggested a different cutoff values for AFP. At a cutoff value >100ng/mL the diagnostic sensitivity was 66.6%, specificity was 93.6% and at a cutoff 200ng/mL low sensitivity (32.2%) and high specificity (100%) were recorded. When the cutoff increase >400 ng/mL, the sensitivity decreased to 13.5%. When increasing the cutoff of serum AFP, the sensitivity decrease and the specificity increase but at the cost of a progressive increase in the false-negative results (El-Assaly et al., 2008).

From (ROC) curve analysis, we found that the best cut-off level of *MicroRNA-215* for discriminating HCC patients versus control groups was 2.30. This had a diagnostic sensitivity of 80%, specificity 96.7%. For discrimination between cirrhosis and HCC groups regarding *microRNA-215*, a discrimination was displayed with 78.3% sensitivity, 88.0% specificity at the best cutoff value of ≤ 1.90. The AUC was 0.87 (p-value < 0.001).

When we combined both microRNA-215 and AFP to discriminate between control and cirrhosis groups, the ROC analysis showed 76.0% sensitivity, 81.7% specificity at the best cutoff value of ≥ 0.56. The AUC was 0.85. Between cirrhosis and HCC groups, the ROC analysis showed a sensitivity of 78.3%, a specificity of 90.7% at the best cutoff level of ≥ 0.47. The AUC was 0.85 (p-value < 0.001).

In conclusion, *MicroRNA-215* was proved to be significantly lower in patients with HCC compared to cirrhotic patients and control group. This marker might be used as a potential marker for HCC diagnosis. An observation worth further investigation is that the combined use of (*microRNA-215* and AFP), increase the diagnostic performance for detection of HCC which suggested that the inclusion of *MicroRNA-215* to the current standard tests for early detection of HCC may improve the ability to identify patients who might be missed by current diagnostic methods and thus might provide a better therapeutic outcome. Moreover, this *miRNA* have an elevated advantage over traditional biomarkers since plasma *miRNAs* are very stable and remaining for long time and their determination techniques are easy to perform. Additionally, their detection in plasma can clearly define disease progression.
